# Denominator Matters: Comparing the Impact of Estimated Resident Population Versus Medicare Enrolment Population on Healthcare Utilisation Analyses

**DOI:** 10.1002/hpja.70069

**Published:** 2025-07-06

**Authors:** Imaina Widagdo, Anna Kemp‐Casey, Gereltuya Dorj, Andre Andrade, Nicole Pratt

**Affiliations:** ^1^ Quality Use of Medicines and Pharmacy Research Centre, Clinical and Health Sciences University of South Australia Adelaide Australia

**Keywords:** denominator, health service, healthcare utilisation, resource allocation

## Abstract

**Issue Addressed:**

The Australian Bureau of Statistics (ABS) Estimated Resident Population (ERP) and the Medicare Enrolment population are commonly used denominators in calculating healthcare utilisation rates. The ERP is an estimate of all usual residents of Australia, even those ineligible for Medicare, while the Medicare population is limited to those eligible for Medicare. However, many researchers may be unaware of these differences, which can lead to inappropriate denominator use, misinterpretation of utilisation rates and suboptimal resource allocation. This analysis compared differences in utilisation rates based on whether ERP or Medicare populations were used as denominators.

**Methods:**

We compared General Practitioner (GP) utilisation rates across age groups, sex and jurisdictions in Australia. Data on GP attendance (Medicare item 23) and published per capita utilisation rates were obtained from the Medicare Statistics website. Per capita service use rates were recalculated using the ERP at June 2022 as the denominator, with the published number of claims as the numerator. The study analysed data from the financial year 2021–2022.

**Results:**

The ERP included 26 million people, while the Medicare population was 26.2 million. Nationally, GP attendance rates were about 2.3% higher using the ABS ERP than using the Medicare population. However, discrepancies varied by age and jurisdiction. In the ACT, ERP‐based rates were around 16% lower than Medicare for persons aged 15–24, whereas in the NT, ERP‐based rates for females aged 85+ were 21% higher than Medicare rates.

**Conclusion:**

Nationally, differences between healthcare utilisation rates calculated using ABS ERP versus Medicare enrolment data were minimal. ERP‐based rates were lower for younger adults, while Medicare‐based rates were lower for older adults.

**So What?:**

Our findings emphasise the importance of carefully selecting and reporting denominators, considering their relevance to the population targeted by the service to ensure meaningful interpretation.

## 1 Introduction

Accurate measures of healthcare utilisation are critical for informing strategies to improve service accessibility, target at‐risk populations and evaluate the impact of public health interventions. Raw data on service use, such as number of General Practitioner (GP) attendance claims, lacks context. Rates and ratios, calculated by dividing total service use by the target population, offer valuable context for understanding coverage, healthcare use trends, and variations by location, age and sex. Selecting the appropriate denominator for rate calculations enables a clearer understanding of healthcare utilisation across different populations, which is essential for evidence‐based health policy and planning.

In Australia, two population‐based denominators are available: the Estimated Resident Population (ERP) from the Australian Bureau of Statistics (ABS) [1] and Medicare enrolment population [2, 3]. ERP data, publicly accessible via the ABS website, reflects the total Australian population, regardless of Medicare eligibility. It is updated quarterly and derived from the Census, birth and death registrations and migration data, and is subject to revision [3]. Medicare enrolment data includes individuals enrolled at the end of each reporting month and represents an administrative record of people enrolled in Medicare. These differences in population scope may lead to the underestimation of service needs or the misidentification of overuse in certain groups. A previous study has compared ERP and Medicare enrolment denominators in the context of HPV vaccination coverage, finding slightly lower ERP‐based rates among young people aged 12–26 years [4]. It was limited to specific age groups (12–26 years). To our knowledge, no previous study has directly compared ERP and Medicare across all age groups to provide a broader assessment on the impact of denominator selection. This issue has become increasingly relevant given Australia's rising migration level. It is also noted that primary care is predominantly covered by Medicare, but hospital services may include individuals not captured in either dataset, such as tourists who may or may not be eligible for Medicare depending on their country of origin, and short‐term visitors may not qualify for inclusion in the ERP [3], adding complexity to denominator selection.

This descriptive analysis aims to examine how the choice of denominator—ERP or Medicare enrolment—affects healthcare utilisation rates, with a focus on understanding the magnitude and direction of differences and to explore the implications for health policy and planning.

## 2 Methods

To demonstrate the impact of denominator selection, we analysed GP attendance claim rates using the Medicare Benefits Schedule (MBS) item 23—a standard GP consultation service less than 20 min long—as it is the most commonly claimed MBS item. Data on the number of GP attendances and per capita rates for the financial year 2021–2022 were obtained from the publicly available Medicare Statistics website. While any financial year would illustrate potential differences between denominators, we selected the 2021–2022 financial year as it was the most recent period for which both Medicare and ERP data were available at the time of writing and could be aligned for comparison. The total Medicare population was derived from the published number of services and per capita rates to enable comparison with the ERP.

As described in the Medicare Statistics methodology, per capita rates (per 100 000 population) are calculated by dividing the number of processed services in a given month by the number of individuals enrolled in Medicare at the end of that month [2]. For comparison, per capita service use rates (Medicare‐based rates) were recalculated using ERP at June 2022 as the denominator, using the published number of GP attendances as the numerator. Percentage differences from Medicare‐based rates were calculated using the following formula:

$$\frac{\left(\mathrm{ERP}\;\text{based rates}-\text{Medicare based rates}\right)}{\text{Medicare based rates}}*100$$

The analysis included individuals with specified age and sex from the eight Australian states and territories, based on both ERP and Medicare data.

## 3 Results

A comparison of population denominators by age group (Table 1) showed that ERP was higher than Medicare in younger adults (15–34 years) but lower in most older groups. The ERP included 26 million people, slightly lower than the Medicare population of 26.2 million, a difference of 261 427 (1.0%).

**TABLE 1:** Comparison of Estimated Resident Population (ERP) at June 2022 and derived Medicare Population by age group, 2021–2022.

Age group	ERP	Medicare population (derived)	Difference (ERP—Medicare)	
			Number	Percent
0–4	1 512 822	1 478 761	34 061	2.3%
5–14	3 250 285	3 324 621	−74 336	−2.2%
15–24	3 171 083	3 043 631	127 452	4.2%
25–34	3 741 527	3 538 840	202 687	5.7%
35–44	3 598 551	3 764 973	−166 422	−4.4%
45–54	3 275 376	3 470 174	−194 798	−5.6%
55–64	3 026 261	3 125 210	−98 949	−3.2%
65–74	2 447 123	2 508 579	−61 456	−2.4%
75–84	1 438 011	1 442 044	−4033	−0.3%
85+	547 102	572 734	−25 632	−4.5%
All	26 008 141	26 269 568	−261 427	−1.0%

**FIGURE 1:**
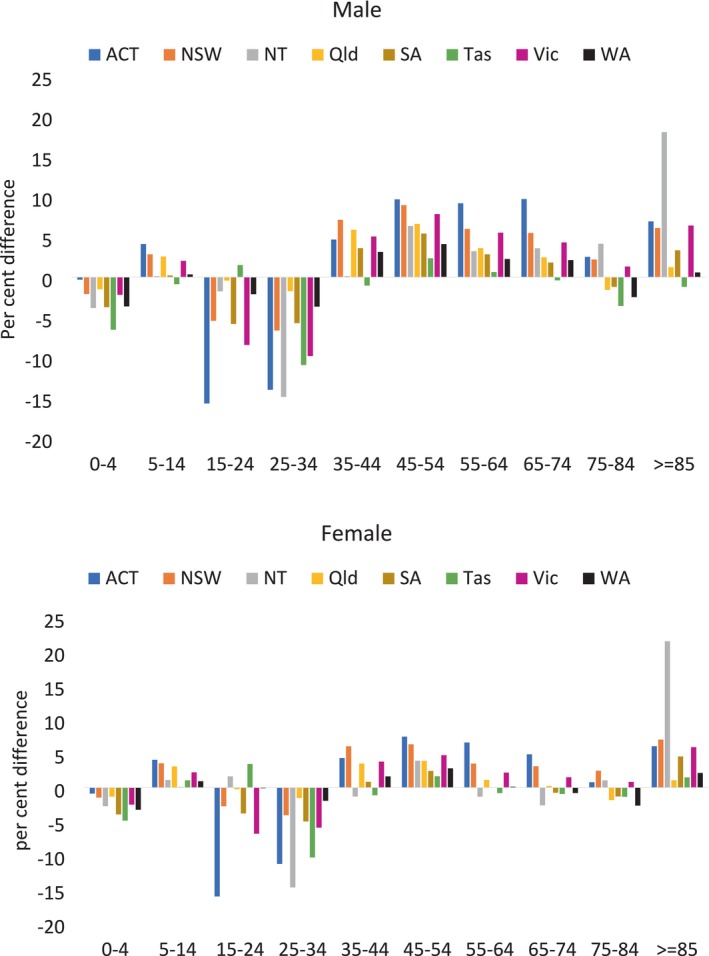
MBS item 23 utilisation percentage difference between Estimated Residents Population rates and Medicare enrolment population rates by age group, sex and jurisdiction, 2021–2022.

In 2021–2022, there were 71 255 099 GP attendance claims under item 23, with Medicare‐based rates at 271 246 per 100 000 population and ERP‐based rates at 277 415 per 100 000—a minimal difference of 2.3% nationally. However, more substantial differences emerged when disaggregated by age, sex, and jurisdiction (Figure 1, Table S1).

For the youngest age group (0–4 years), ERP‐based rates were consistently lower than Medicare‐based rates across all states, with the largest difference in Tasmania among males (6.5%). In the 5–14 age group, ERP‐based rates were generally slightly higher than Medicare rates, with the greatest difference in the ACT at ~4% for both males and females. Among young adults (15–24 and 25–34), ERP‐based rates were reasonably lower, with the most substantial difference in the ACT (16% lower than Medicare rates). For age groups between 35–44 and 75–84 years, ERP‐based rates were typically higher than Medicare rates, with differences remaining modest (under 10%) and decreasing with age. The most marked differences appeared in those aged 85 and older, with ERP‐based rates for females in the Northern Territory being 21% higher than Medicare rates.

## 4 Discussion

At the national level, the ERP was slightly lower than the Medicare population in 2021–2022 (26 vs. 26.2 million), a difference of 1%. This translated to only a 2.3% difference in GP attendance rates nationally. However, as shown in Table 1 and Figure 1, these differences varied considerably across age groups and jurisdictions, whereas differences by sex were minimal. The findings highlight that even small national‐level differences can obscure more substantial variation at the subpopulation level. These differences may be explained by several factors. Among newborns, the higher Medicare rates could be due to delays in Medicare registration which result in lower population numbers in Medicare enrolment at the time of reporting compared to ERP (Table 1), as ERP incorporates birth data in its calculation, although these are subject to revision due to registration delays. For the 85‐and‐older age group, delays in deregistration after death may inflate Medicare enrolment numbers (Table 1), while ERP accounts for mortality rates. In the young adult age group, ERP may include international students or temporary visa holders who are not eligible for Medicare [3], which could explain the lower ERP rate. This finding aligns with the results from a previous study comparing ERP and Medicare denominators for HPV vaccination coverage, which also reported lower ERP‐based rates among people aged 12–26 years [4]. For middle‐aged adults (35–64 years), Medicare rates may be lower than ERP rates due to the inclusion of individuals covered under Reciprocal Health Care Agreements, who may not qualify for ERP if their expected stay is less than 12 months [5]. Some of the larger differences observed in jurisdictions such as the ACT and NT may reflect their smaller population sizes and unique population profiles (e.g., higher proportions of transient or non‐resident individuals), which can magnify the impact of denominator differences. These variations reflect the fundamental differences between the two denominators: ERP represents the usual resident population, calculated using Census data and updated with births, deaths and migration data [3], while Medicare enrolment is subject to delays and eligibility restrictions.

These findings highlight the impact of denominator choice in healthcare utilisation analyses and the importance of aligning the choice with the purpose of the analysis. If the aim is to understand uptake of Medicare‐subsidised services, the Medicare‐enrolled population is more appropriate; for broader public health analyses, the ERP may be more suitable. Using ERP may underestimate Medicare‐subsidised service needs among young adults by including non‐Medicare‐eligible individuals like international students. Conversely, Medicare enrolment data may underestimate potentially inappropriate or high‐risk medicine dispensing in older populations, as the denominator may be inflated (Table 1) by the inclusion of recently deceased individuals. These discrepancies have significant implications for health promotion, as inaccurate denominators can lead to misallocation of resources, hinder targeted interventions, and obscure service gaps in at‐risk populations, thus reducing the effectiveness of health promotion efforts. Importantly, both ERP‐ and Medicare‐based rates should be interpreted as indicative estimates, and users should be aware of their respective limitations when applying them for service planning or policy decisions. This analysis was limited to publicly available ERP and Medicare data, which include only age, sex, and State or Territory aggregates. Ethnicity, Indigenous status, and more detailed geographical area data are not readily available in Medicare data.

In New Zealand, the Health Service User (HSU) dataset, which is a combination of datasets across the health sector and recently reviewed by Stats NZ [6], offers a dynamic and activity‐based approach to defining denominator populations for healthcare utilisation analyses. The HSU dataset includes individuals who have interacted with the health system within a given year, regardless of residency status, providing a more accurate reflection of actual service users compared to the total resident population. This approach enhances alignment with healthcare utilisation data, particularly in identifying active health system users and capturing service‐specific trends. However, a key limitation of the HSU dataset is its exclusion of individuals who do not access healthcare services, potentially obscuring patterns of inactive healthcare users and creating gaps in equity analyses. Additionally, there is known undercount and misclassification of Māori in HSU data [7], as well as concerns around inclusion and eligibility for Pacific populations living in New Zealand [8]. Similar limitations exist in Australia, where the under‐identification of Aboriginal and Torres Strait Islander peoples in administrative datasets such as Medicare and hospital data has been widely acknowledged [9].

Similar denominator challenges have also been observed in the United Kingdom. National Health Service (NHS) Digital's 2022 spotlight report highlighted that GP patient registration data exceeds official population estimates, with discrepancies attributed to delayed de‐registration, duplicate records, and inclusion of individuals who have moved or are deceased [10].

These limitations highlight the need to carefully select denominators that align with the specific context and objectives of the analysis to ensure accurate and equitable insights into healthcare utilisation.

The availability of whole‐of‐population Australian linked datasets provides valuable opportunities for identifying fit‐for‐purpose denominator populations. For example, the ABS Person Level Integrated Data Asset (PLIDA) [11], which integrates Medicare data, death registrations and migration records, enables a more precise estimation of population subgroups eligible for healthcare services. Leveraging such integrated datasets could improve the validity of healthcare utilisation analyses, reduce biases introduced by denominator selection, and support evidence‐based health policy and planning. However, their use is constrained by restricted access, ethics and governance approvals, and processing timelines. Furthermore, PLIDA does not include publicly available denominator data. In contrast, ERP and Medicare data are readily accessible. Therefore, we recommend that researchers and health service planners align denominator selection with the intended purpose of the analysis, understand the limitations of each denominator source when interpreting results and advocate for greater investment in the use and accessibility of integrated data sources to support more accurate, equitable and policy‐relevant health system insights.

## 5 Conclusion

At the national level, healthcare utilisation rates calculated using ABS ERP and Medicare enrolment data showed only modest differences overall, but notable age‐related variation. ERP‐based rates were lower for younger adults, while Medicare‐based rates were lower for older adults. Our analyses highlight the complexity of denominator selection, as it can meaningfully affect rate interpretation and each data source has different strengths and weaknesses. Accurately measuring healthcare utilisations requires balancing inclusivity with specificity in the definitions or data sources used for both the numerator and denominator to support effective policy planning. Future work should explore methods for real‐time validation of denominator data and expand access to integrated datasets, such as PLIDA, to improve accuracy, equity insights and policy relevance in health planning.

## Conflicts of Interest

The authors declare no conflicts of interest.

## Supporting information


**Supplementary Table S1.** Comparison of GP Attendance (MBS Item 23) Rates Using Medicare‐Enrolled and ABS ERP Populations by Age, Sex, and State, FY2021–22.

## Data Availability

The data that support the findings of this study are available in Medicare Statistics at http://medicarestatistics.humanservices.gov.au/statistics/mbs_item.jsp. These data were derived from the following resources available in the public domain:—Medicare Statistics, http://medicarestatistics.humanservices.gov.au/statistics/mbs_item.jsp‐ Australian Bureau of Statistics, https://www.abs.gov.au/statistics/people/population/national‐state‐and‐territory‐population/mar‐2024.
